# An Intriguing Case Report of Functional Mitral Regurgitation Treated With MitraClip

**DOI:** 10.1097/MD.0000000000000608

**Published:** 2015-05-22

**Authors:** Vincenzo Duino, Luigi Fiocca, Giuseppe Musumeci, Emilia D’Elia, Mauro Gori, Elisa Cerchierini, Orazio Valsecchi, Michele Senni

**Affiliations:** From the Cardiovascular Department (VD, LF, GM, ED, MG, OV, MS), Anesthesiology Department (EC), Hospital Papa Giovanni XXIII, Bergamo, Italy.

## Abstract

Functional mitral regurgitation (FMR) is frequent in patients with heart failure (HF). It develops as a consequence of left ventricle (LV) geometry alterations, causing imbalance between increased tethering forces and decreased closing forces exerted on the mitral valve apparatus during systole.

FMR is known to change at rest and during effort, due to preload–afterload changes, myocardial ischemia, and/or LV dysfunction. Despite optimized medical therapy, an FMR can be responsible of shortness of breath limiting quality of life and decompensation. In this report, we present a case of dynamic FMR treated with MitraClip.

MitraClip implantation is a successful and innovative opportunity for HF patients with FMR.

## INTRODUCTION

Functional mitral regurgitation (FMR) is a common finding in patients with heart failure (HF), it develops as a consequence of left ventricle (LV) geometry alterations, causing imbalance between increased tethering forces and decreased closing forces of mitral valve apparatus during systole.^1,2^ One of the main characteristics of FMR is its variability at rest or during effort related to preload–afterload changes, myocardial ischemia, and/or LV dysfunction.

In this article, we report a challenging case of MitraClip implantation in a setting of dynamic FMR (Carpentier Type IIIb) in HF with reduced ejection fraction (HFrEF).

## CASE REPORT

In July 2010, a 63-years-old woman affected by diffuse large B-cell lymphoma started chemotherapy with R-CHOP14 (rituximab-cyclophosphamide-doxorubicin-vincristine-prednisolone). A baseline transthoracic echocardiogram (TTE) was normal. In August 2011, she was referred to our HF clinic because the TTE documented an initial LV dysfunction (LV ejection fraction [LVEF] 45%), moderate mitral regurgitation (MR), and increased systolic pulmonary artery pressures (SPAP 50 mmHg). In the hypothesis of chemotherapy-induced cardiotoxicity, betablocker and angiotensin converting enzyme (ace)-inhibitor were started. In the meantime, total regression of lymphoma was documented.

From January 2012, symptoms gradually worsened with shortness of breath in New York Heart Association (NHYA) class III. A TTE documented a further decrease of LVEF (30%), moderate FMR, massive tricuspidal regurgitation (TR), and dilated inferior vena cava. In November 2012, a dobutamine-stress-echocardiography (DSE) showed no myocardial ischemia nor contractility reserve. In March 2013, a left heart catheterization evidenced normal coronary vessels, LV dilatation with markedly depressed LVEF, and increased LV filling pressure (20 mmHg). A 6-minute walking test documented low performance (120 m), but no peripheral desaturation. Betablocker and ace-inhibitor were titrated and diuretics added.

**FIGURE 1:**
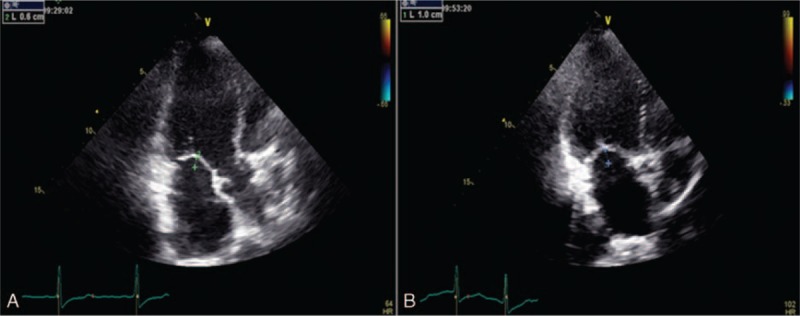
Increased coaptation depth during stress: 10 mm (B) compared to baseline 6 mm (A), due to the rise in the forces of tethering.

**FIGURE 2:**
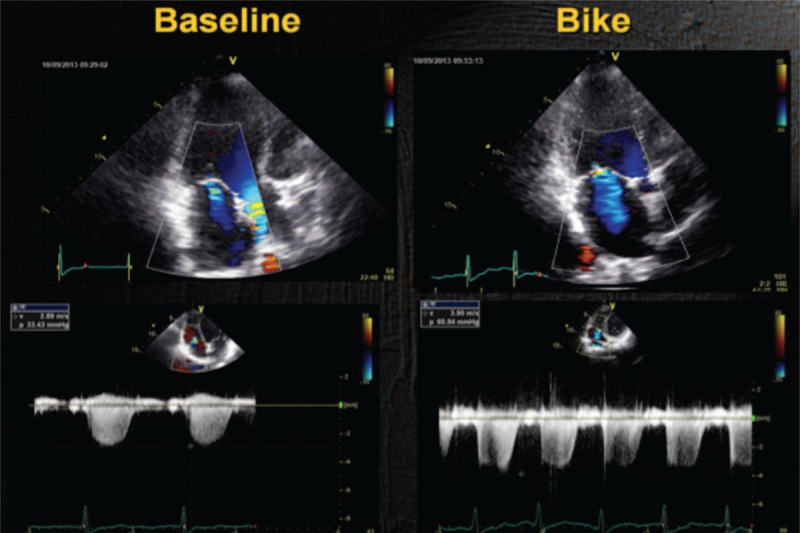
Baseline: mild MR. Exercise: increase in the degree of MR and SPAP. MR = mitral regurgitation, SPAP = systolic pulmonary artery pressures.

**FIGURE 3:**
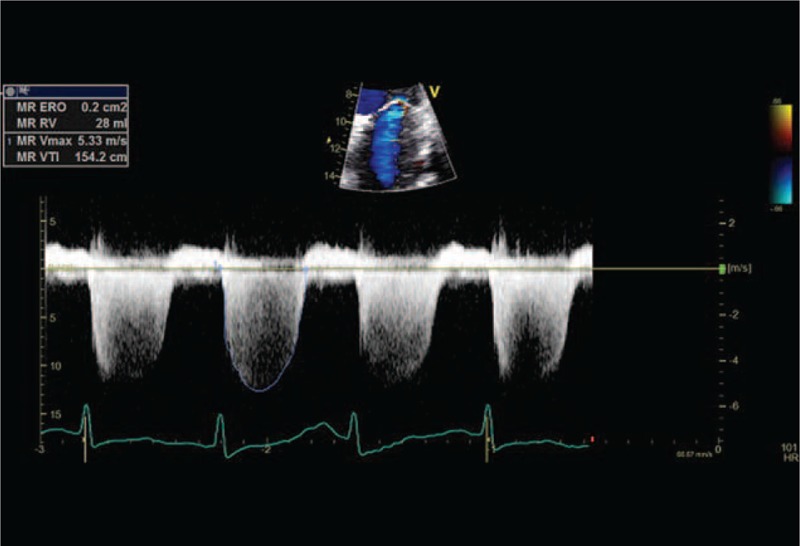
MR during cycle-stress-echocardiography (EROA = 0.2 cm^2^, RV 28 mL). EROA = effective regurgitant orifice area, MR = mitral regurgitation, RV = regurgitant volume.

**FIGURE 4:**
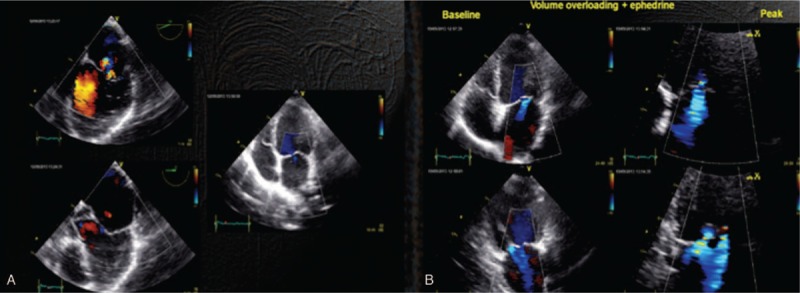
Echo lab. (A) TEE and TTE demonstrate minimal regurgitation. (B) After handling the load (saline and ephedrine) increase in the regurgitation with very obvious area of flow convergence. TEE = transesophageous echocardiogram, TTE = transthoracic echocardiogram.

**FIGURE 5:**
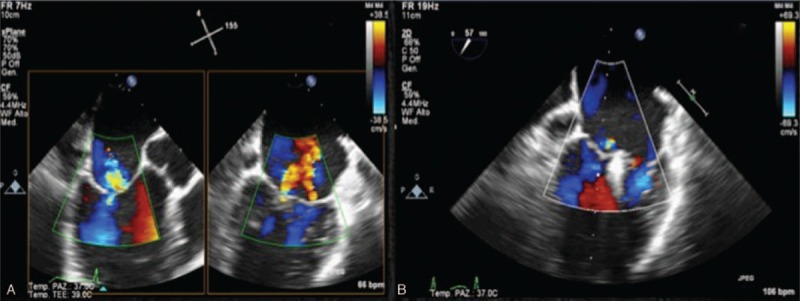
Cath-lab. (A) Preimplant: sufficient evidence of regurgitation and convergence area after volume load (ephedrine and norepinephrine). (B) After implant: clip in place. Insignificant regurgitation also in the course of norepinephrine administration.

**FIGURE 6:**
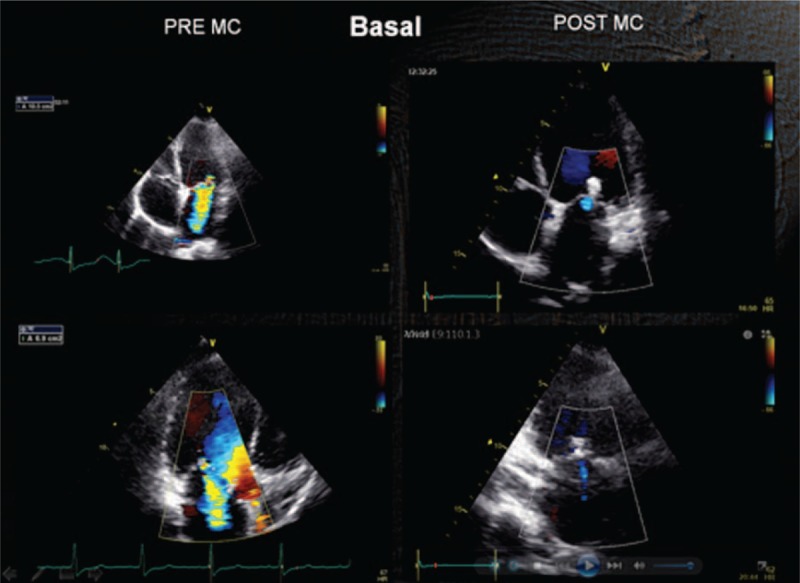
TTE at baseline preimplantation and postimplantation: after implantation regurgitation is almost absent. TTE = transthoracic echocardiogram.

In May 2013, the patient still complained for shortness of breath (NHYA class III); the TTE showed a further LV dilatation with LVEF 45%, moderate FMR (vena contracta [VC] 4 mm), and moderate TR. Medical therapy was optimized with ivabradine. As the woman still complained dyspnea at rest, in the hypothesis of a dynamic FMR, a cycle-stress-echocardiography was performed. It was stopped at 50 watts for dyspnea, without signs of ischemia. A consistent increase of FMR, from moderate to severe, was observed (effective regurgitant orifice area [EROA] 0.1 > 0.2 cm^2^, regurgitant volume [RV] 13 > 28 mL, color area of MR 6.3 > 11.2 cm^2^, mitral annulus 27 > 32 mm, VC 4 > 6 mm), probably due to an increase in the tethering forces, with a significant increase in SPAP (33 > 61 mmHg) (Figures 1–3). Basal LVEF was 45% (left ventricle end diastolic diameter/systolic [LVDV/S] 120/66 mL) and 51% (LVDV/S 127/62 mL) during stress. Surprizingly, 2 days after the transesophageous echocardiogram (TEE) showed only a mild MR. Considering the impact of the preload/afterload variations in the quantification of an FMR, the patient was investigated with physiological solution (1000 mL in 15 minutes), and ephedrine (10 mg) infusion tests with marked worsening of the FMR that appeared suitable for a MitraClip procedure (Figure 4). Before taking a decision, the case was also discussed with surgeons and cardio-anesthesiologists who declined to treat the patient due to the high surgical risk. Since we still considered exertional increase in MR the main cause of symptoms, we decided to undergo to MitraClip implantation. The day of the intervention, on general anesthesia, TEE showed mild FMR without evidence of an adequate convergence area also under physiological solution and ephedrine infusion. Only under noradrenalin infusion severe MR appeared and grasp of the leaflets in the correspondence of the regurgitant jet became feasible. Before the release of the clip, noradrenalin was infused again with evidence of only mild MR (Figure 5). A predischarge TTE confirmed the success of the procedure with trivial MR left (Figure 6).

**FIGURE 7:**
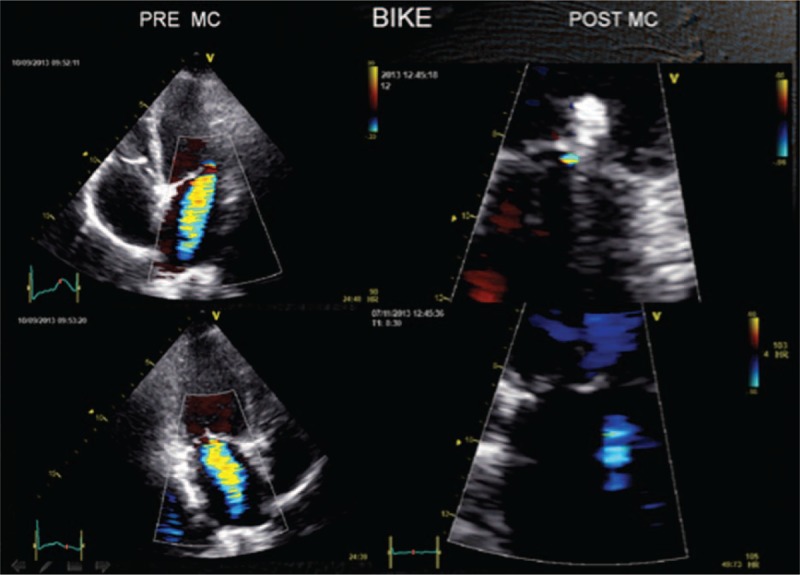
Exercise Doppler echocardiography: on the left, preimplant, moderate-to-severe MR. On the right, postimplant, MR now is negligible. MR = mitral regurgitation.

The follow-up assessment at 40 days showed improvement in 6MW distance (120 > 150 m) and a better result of the echo-bike (up to 100 W) with no MR increase during effort (Figure 7). TTE at 8 months confirmed the success of the implantation, with a significant improvement of the functional status (NHYA I). Informed consent was given by the patient for using her clinical data.

## DISCUSSION

The Euro Heart Survey of the European Society of Cardiology showed that MR is present in 80% of HF patients, and that in 50% of them it is greater than moderate.^3^ Significant changes of the degree of MR in relation to physical activity are not predictable nor related to the degree of MR at rest^4,5^ but they are associated to both morphofunctional changes of the mitral valve apparatus and the dynamic variations of ventricular dyssynchrony.^6,7^ Although some patients have little dynamic variations, about 30% of them show an important increase in MR and SPAP during exercise. Dynamic change of MR in the case of severe LV dysfunction and little contractility reserve is crucial in the pathogenesis of impaired exercise tolerance. The increase in afterload and in the MR degree determines a reduction in anterograde flow and an increase in SPAP clinically manifested by the onset or worsening dyspnea during physical activity^8,9^ or acute pulmonary edema in adverse hemodynamic conditions.^10^ In HF with preserved ejection fraction, dynamic increase in MR and SPAP have been demonstrated during decompensation.^11^

Dynamic changes of MR can be finely investigated with exercise echocardiography, which exactly reproduces the physiological conditions. DSE is not adequate in this setting as it usually determines a reduction in MR.^10^

An increase in EROA ≥ 0.13 cm^2^ has an important prognostic value, as well as an increase in the RV and SPAP,^9^ since they contribute to a worse prognosis with increased mortality and morbidity.^12^

The identification of dynamic components in the development of severe MR during exercise can be helpful to guide surgical strategy in ischemic patients candidate for coronary surgery, in order to avoid progressive LV dilation or dysfunction. Less obvious might seem the meaning of a dynamic MR in a patient not candidate for surgery. It seems arduous to consider a surgical correction of an isolated mild to moderate FMR at rest, moreover in patients at high surgical risk. Nevertheless, we often face patients as the one we reported: despite optimized drug therapy, symptoms significantly limit quality of life and frequent episodes of decompensation occur.

Nowadays, the possibility to correct MR with percutaneous implantation of the MitraClip is a valid alternative to surgery, in selected cases.^12,13^ The procedure is currently consolidated, with widely demonstrated safety and efficacy in more than 15,000 cases in the world. In current clinical practice, this procedure is mainly performed in patients with HFrEF and FMR, usually older than 70-years old, with comorbidities and high surgical risk.^14–17^ Data from real-world registries show the effectiveness of MitraClip implantation in terms of improvement of exercise tolerance and quality of life, reduction of rehospitalization for HF, and a trend in mortality reduction compared with medical therapy.^14,18^ Anyway, ongoing randomized trials comparing MitraClip with standard medical therapy (A Randomized Study of the MitraClip Device in Heart Failure Patients With Clinically Significant Functional Mitral Regurgitation; Cardiovascular Outcomes Assessment of the MitraClip Percutaneous Therapy for Heart Failure Patients with Functional Mitral Regurgitation Trial) will definitely assess the exact clinical role of the procedure results. The case we presented has peculiar pathophysiological and technical aspects: recognition of the increased effort related to MR and SPAP as the main cause of symptoms and possible poor outcome, decision to correct the dynamic valvular defect with MitraClip implantation, and difficulty to obtain (due to general anesthesia) an accurate view of the regurgitant jet. Collaboration with the cardio-anesthesiologist during the procedure was crucial, and the use of noradrenaline was effective in evoking a degree of MR similar to the one obtained during echo-bike. This case represents, in our opinion, a clear example of the heart team importance in a very challenging HF setting. To our knowledge, this is the first case in literature of a successful MitraClip implantation in FMR whose dynamic appearance significantly limits quality of life.
